# On Comparison of Heat Treated and Non-Heat-Treated LOM Manufactured Sample for Poly(lactic)acid: Mechanical and Morphological View Point

**DOI:** 10.3390/polym14235098

**Published:** 2022-11-24

**Authors:** I. Singh, S. Kumar, S. S. R. Koloor, D. Kumar, M. Y. Yahya, J. Mago

**Affiliations:** 1 Department of Mechanical Engineering, CT University, Ferozepur Rd, Sidhwan Khurd, Ludhiana 142024, India; 2 Institute for Structural Engineering, Department of Civil Engineering and Environmental Sciences, Universität der Bundeswehr München, Werner-Heisenberg-Weg 39, Neubiberg, 85579 Munich, Germany; 3 Centre for Advanced Composite Materials, School of Mechanical Engineering, Faculty of Engineering, Universiti Teknologi Malaysia, Johor Bahru 81310, Johor, Malaysia; 4 Center for Automotive Research and Tribology, Indian Institute of Technology Delhi, Hauz Khas, New Delhi 110016, India

**Keywords:** FDM printing, LOM manufacturing, heat treatment, compressive strength, SEM analysis, comparative analysis

## Abstract

This work reports the comparison of heat-treated and non-heat-treated laminated object-manufactured (LOM) 3D-printed specimens from mechanical and morphological viewpoints. The study suggests that heat treatment of the FDM-printed specimen may have a significant impact on the material characteristics of the polymer. The work has been performed at two stages for the characterization of (a) non-heat-treated samples and (b) heat-treated samples. The results for stage 1 (non-heat-treated samples) suggest that the infill density: 70%, infill pattern: honeycomb, and six number of discs in a single LOM-manufactured sample is the optimized condition with a compression strength of 42.47 MPa. The heat treatment analysis at stage 2 suggests that a high temperature: 65 °C, low time interval: 10 min, works equally well as the low temperature: 55 °C, high time interval: 30 min. The post-heat treatment near Tg (65 °C) for a time interval of 10 min improved the compressive strength by 105.42%.

## 1. Introduction

Polymer and polymer-based composite materials found many interests in advanced industrial applications due to their innovative design and mechanical features. Various advanced manufacturing processes including mixing, forming, laminating, etc., are practiced to overcome the mechanical challenges for the optimum fabrication of polymer structures [1,2,3]. Among these, the additive manufacturing of polymeric material through the FDM route is one of the most explored routes. The past decade has seen an exponential uprise of FDM-related work. The application area for the material and design-specific FDM-printed parts has also seen strong growth. Various studies in past have reported mechanical- and morphological-specific improvement of FDM-printed parts when the parts are post-processed by selecting the suitable route [4,5,6].

One such study performed by Yarahmadi et al. [7] heat treated polyvinyl chloride (PVC) for the selected range of temperature between 55 °C to 165 °C for a period of 1 h to 143 days. The work observed a decrease in break elongation with an increase in the temperature above 55 °C and with increased aging time. Fouad et al. [8] observed the effect of post-heat treatment of ultra-high molecular weight polyethylene (UHMWPE) polymeric material at 50 °C, 80 °C, and 100 °C for 2 to 4 h. The work observed an improvement in the fracture strength, modulus, and yield strength of the tensile specimen significantly in the range of 11 to 25% for different material characteristics. Pagno et al. [9] studied the effect of heat treatment on Poly (butylene adipate-co-terephthalate) (PBAT) and polycaprolactone (PCL) reinforced matrix for membrane application in drug removal. The study improved the retention capacity of electro-spun-produced membranes by 67% with heat treatment.

Chalgham et al. [10] worked on PLA material and observed the mechanical properties of PLA before and after heat treatment. The heat treatment for the polymeric samples had been performed at 75 °C for bending the samples to give them the perfect shape for finger orthosis. The results of the study suggested that heat treatment of samples had led to a change in the geometry of the 3D-printed samples for orthosis to the required shape without decreasing the maximum force. Jayanth et al. [11] reported the chemical treatment of ABS after FDM printing. The chemical treatment of the FDM-printed samples led to a decrease in the tensile strength of the ABS samples when chemically treated. The surface characteristics of the samples were improved, such as surface finish. Storck et al. [12] tested different materials of FDM printing for elevated temperature applications such as space and satellite purposes. The study had given the exposure of −40 °C to +80 °C to 3D-printed samples by selecting cycles of 90 min. The samples of PLA material were observed to be stable for the varying temperature cycles whereas, other materials lost their dimensional and mechanical characteristics.

Zhao et al. [13] explored the post-heat treatment process effect for polyether ether ketone (PEEK). The work selected a temperature of 150 °C to 250 °C (above glass transition temperature Tg) for heat treatment and a time interval of 2 h. The tensile strength for the PEEK samples was observed to be increasing with increasing temperature. Wang and Zou [14], also observed the effect of heat treatment on short and continuous fiber-reinforced PEEK material matrix. The study highlighted that the heat treatment process improved the layer adhesion and diffusion of the adjacent layers for FDM-printed PEEK reinforced matrix leading to better mechanical strength. Wach et al. [15] explored the thermal annealing process for PLA matrix to improve the crystallinity degree. The study observed an 11 to 20% improvement in the PLA strength when annealed over the Tg of the material.

Li et al. [16] worked on the sanding or plasma treatment of the 3D-printed PEEK, PEEK/CF material. The study indicated a significant increase in the shear strength of the lap-jointed 3D-printed workpiece and other surface characteristics of the samples when treated with sanding or plasma treatment before joining the samples for the lap joint. Thermal post-treatment of polymeric samples may result in part deformation or surface cracks due to thermal shocks developed over the outer surface. Cerezo et al. [17] explored heat treatment using a ceramic powder mold which reduced 80% to 90% chances of deformation in geometry, especially in the length of the FDM-printed samples of ABS. Akhoundi et al. [18] studied the impact of the heat treatment annealing process on FDM-printed parts of PLA. The results of the study suggested up to a 32% increase in the mechanical strength for the heat-treated samples depending on various selected temperatures for heat treatment.

One similar study performed by Guduru and Srinivasu [19] explored the heat treatment process on PLA/CF reinforced FDM-printed samples. The study reported a significant increase in the tensile strength of PLA/CF material for optimized samples using chemical treatment (80 MPa) and heat treatment (74 MPa) route than normal PLA/CF FDM-printed matrix (70 MPa). Researchers have made effort toward the 4D printing of PET-G [20] and tried to give material self-healing characteristics and observed that 75 °C temperature is the optimized range where material exhibits self-healing from bending. Below and above this temperature, the material has shown the least effect on self-healing and shape recovery.

Ehrmann et al. [21] explored the post-heat treatment process for PLA material for different grades and colors of PLA. The reported results suggested that there are contradictions and low reproducibility of the results with the previously reported data. Jayswal and Adanur [22] demonstrated the improved mechanical properties of PLA for textile applications by using a post-heat treatment technique. Furthermore, more crystallinity was found in the case of heat-treated samples compared to samples of non-heat-treated (NHT). McLouth et al. [23] tested atmospheric plasma treatment (APT) on polyetherimide (PEI) and observed 35% improved surface and mechanical characteristics of the samples in terms of bond strength. The study used a higher concentration of oxygen and carboxyl group at the exposed surface to increase the wettability and enhancement of interfacial bonds respectively. It was also observed that the damaged samples of PEI were repaired at a 100% recovery rate by the applied plasma treatment and bonding technique. Moradi et al. [24] annealed the PLA samples using a laser cutting setup by operating the setup at low power conditions. The study reported optimized processing conditions for the post-processing as focal position: 0.53 mm, laser cutting speed: 1.19 mm/s, and laser power 36.49 W. One of the studies related to post-processing has proposed a new method called “Ironing” [25] which deals with heating the stacked layer at a defined level while FDM printing. The results of the study have suggested a 60% improvement in the surface roughness value whereas, the mechanical strength was not explored. Many studies in the past 5 years have been performed dealing with the in-situ treatment or post-treatment of FDM-printed specimens such as using pressure [26], cold vapor post-processing [27], and in-situ heat treatment while FDM printing of PLA specimens [28]. The increased in-situ pressure while FDM printing has tremendously improved mechanical properties by 100 to 150% for PLA samples [26]. The in-situ heat treatment of PLA samples [28] was compared to heat treated sample of a vacuum oven. The results have proved that vacuum oven-based heat treatment improved mechanical strength (tensile strength 90 MPa) better than in-situ treatment (35 MPa). Elsheikh [29] highlighted the scope of bistable morphing composites which have shown promising results for 4D applications. Similar studies have shown recent progress in wood-based composites with their post-treatment processing. The results have highlighted improved mechanical performance of the composite tested for different applications [30]. Table 1 summarizes the literature reviewed and shows the effort made and the findings of the surveyed work.

### Scope of the Present Study

The literature review reveals that there is a scarcity of research data related to Laminated object-manufactured (LOM) FDM-printed samples of PLA. There are also research gaps found in the field of post-treatment of laminated object manufactured (LOM) samples of FDM-printed material and their comparison with the non-heat-treated samples. Therefore, an effort has been made in the direction of FDM printing of multiple discs and arranging them into single specimens using epoxy for LOM manufacturing and characterizing for mechanical and morphological properties of heat-treated (HT) and non-heat-treated (NHT) samples of PLA.

## 2. Materials and Methods

### 2.1. Selection of Material and FDM Printing

In the present study, PLA has been taken as the polymeric material matrix which is procured from eSun manufacturers (Nanshan District, Shenzhen, China, Avaliable online: https://robu.in/; accessed on 25 June 2022). The purchased FDM filament has been used for FDM printing of compression samples as per ASTM D695 standard [31]. Cylindrical samples of dimension 12.7×25.4 mm was successfully FDM-printed based on the L9 design of experimentation (DOE) of the Taguchi orthogonal array (OA) (see Table 2). The samples are printed according to the selected design of experimentation (DOE) for two different stages. The samples are 3D-printed using an FDM printer (City: Navi Mumbai, State: Maharashtra; India; Make: Divide by Zero). A similar testing process on different advanced polymers is practiced elsewhere [32,33]. 

### 2.2. Selected DOE and Stages of Work

For printing the FDM specimen and to explore the effect of heat treatment on the LOM manufactured samples, the work is performed in two stages:

**Stage 1;** (a) FDM printing of samples as per selected DOE, (b) LOM manufacturing of 3D-printed discs, and (c) testing without heat treatment and optimizing the best condition as per ASTM D695 standard of testing for compression properties.

**Figure 1:**
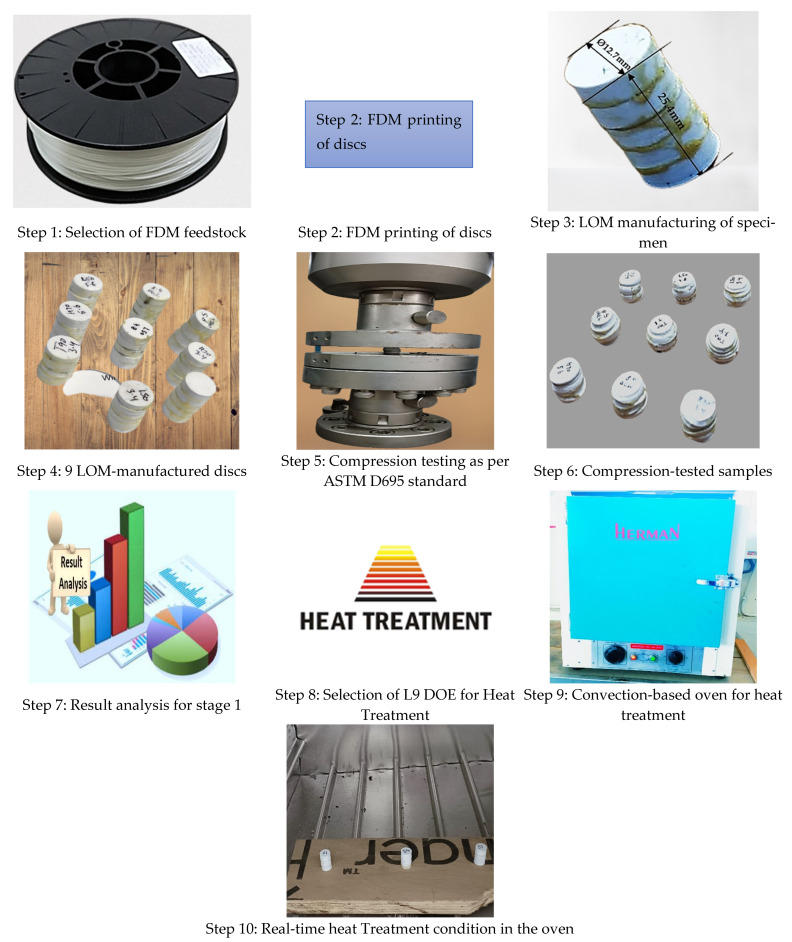
Methodology for the present work to compare non-heat-treated samples and heat-treated samples for compression properties of PLA for stage 1 and stage 2.

**Stage 2;** (a) Selection of DOE for Heat treatment, (b) FDM Printing as per DOE based on the optimized setting of stage 1, (c) LOM manufacturing, and (d) compression testing as per ASTM D695 standards. Figure 1 shows the methodology for the present work to compare non-heat-treated samples and heat-treated samples for compression properties of PLA for stage 1 and stage 2.

Table 2 shows the DOE used for stage 1 to optimize the 3D printing process for compression testing using the LOM manufacturing route. Whereas Table 3 shows the DOE selected based on heat treatment conditions. The DOE contains a parameter for the number of discs, which are joined with each other using epoxy resins after FDM printing. The number of discs was joined to make a single compression sample e.g., in the case of the number of discs is 4 which means the sample is made up of 4 discs, each of thickness of 5.6 mm. the calculation for the total height can be performed using Equation (1).

(1) $$\mathrm{Total}\;\mathrm{height}\;\mathrm{of}\;\mathrm{disc}=25.4\mathrm{mm}\;\mathrm{Each}\;\mathrm{disc}\;\mathrm{thickness}=5.6\mathrm{mm}\;\left(\mathrm{In}\;\mathrm{case}\;\mathrm{number}\;\mathrm{of}\;\mathrm{disc}\;4\right)\;\mathrm{The}\;\mathrm{total}\;\mathrm{thickness}\;\mathrm{of}\;4\;\mathrm{disc}=5.6\times 4=22.4\mathrm{mm}\;\mathrm{Each}\;\mathrm{layer}\;\mathrm{thickness}\;\mathrm{of}\;\mathrm{epoxy}\;=1\mathrm{mm}\;\begin{array}{cc} \mathrm{Total}\;\mathrm{height}\;\mathrm{of}\;\mathrm{disc} & \\ = & \;\mathrm{Each}\;\mathrm{disc}\;\mathrm{thickness}\times \mathrm{number}\;\mathrm{of}\;\mathrm{disc}\;\\ + & \;\mathrm{total}\;\mathrm{layer}\;\mathrm{thickness}\;\mathrm{of}\;\mathrm{epoxy}\;\mathrm{at}\;\mathrm{joining}\;\mathrm{layers} \end{array}\;\mathrm{Total}\;\mathrm{height}\;\mathrm{of}\;\mathrm{disc}=25.4\mathrm{mm}$$

Table 2 which shows the number of discs also mentions each disc thickness for the number of discs to be joined using epoxy. Whereas, in each case, the thickness of the joining layer is constant i.e., 1 mm.

For the present study we have selected a feedstock filament of PLA with a glass transition temperature (Tg) of 64 °C, Therefore, we have selected a range of temperature between 55 °C to 65 °C limits.

### 2.3. Heat Treatment of Specimens and Mechanical and Morphological Testing

The tested samples for compression properties are optimized for the FDM printing as well as for the geometric configuration (number of the disc) of the disc selected in DOE. Further in the second stage to print the samples as per DOE 2, the optimized condition at stage 1 has been chosen and all samples are made using a similar condition as per the optimized setting at stage 1. The samples were then heat treated in the oven at three different temperatures (i) 55 °C, 60 °C, and 65 °C as per the DOE. The samples were cooled in the air after heating for the specified time.

### 2.4. Mechanical and Morphological Property Testing

The prepared samples according to the DOE of different stages 1 and 2 are tested for compression properties by following the ASTM D695 standard. For compression testing standard UTM (Make: Shanta Engineering, Thane, Maharashtra, India) has been used for compression testing with a load cell of 10 KN and an extensometer (Make: AVE639; Dakseries, Thane, Maharashtra, India). The compression analysis has been performed at a testing speed of 20 mm/min.

Further, four different samples (a) optimized samples as per DOE 1 without heat treatment, (b) three samples with heat treatment (i) heat-treated at 55 °C, (ii) at 60 °C, and (c) at 65 °C, are studied for morphological properties using SEM characterization to know the effect of heat treatment on the samples.

## 3. Results and Discussion

### 3.1. Compression Properties for Non-Heat-Treated Specimens (Stage 1)

**Figure 2:**
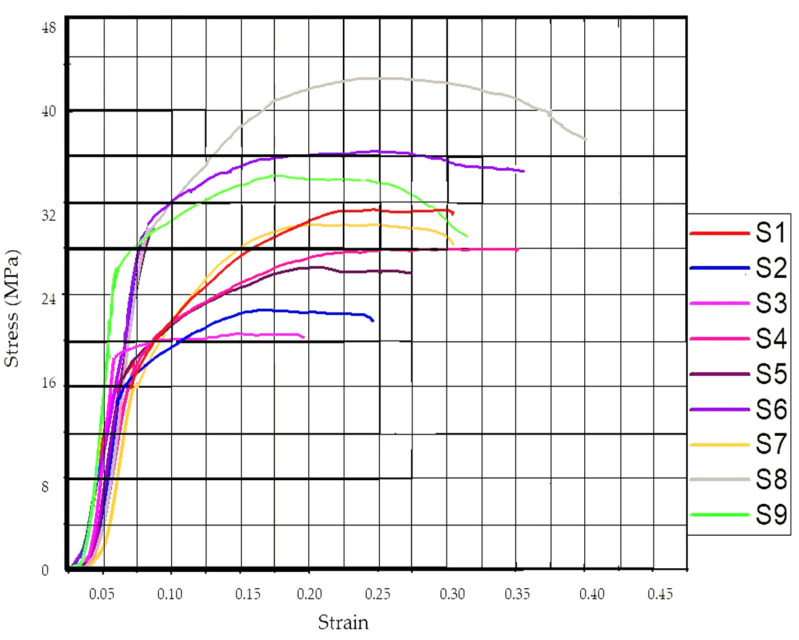
Stress vs Strain for the samples of Table 4.

Table 4 shows the compression properties of the LOM-manufactured samples. From the result table, it may be observed that sample 8 has shown maximum compressive strength of 42.47 MPa with the corresponding strain value of 0.41. Whereas, sample 3rd has shown the least mechanical strength of 21.91 MPa and a corresponding strain of 0.19 MPa. The eighth sample was 3D-printed with an infill density of 70%, infill pattern: honeycomb and the sample contains six discs (maximum) in a single LOM manufactured specimen. The reason for the best characteristics of sample 8 may be due to the reason that every single 3D-printed disc had four upper layers and four lower layers. Sample 8 was made up of a total of 48 layers (upper and lower layers) which contributed to the strength of the sample when compressed. Similarly, sample 3 contains only four discs with 32 upper and lower layers which may have not given resistance to applied compressive force and thus resulted in poor compressive strength. Figure 2 shows the stress vs. strain diagram for the compression-tested samples of stage 1.

**Figure 3:**
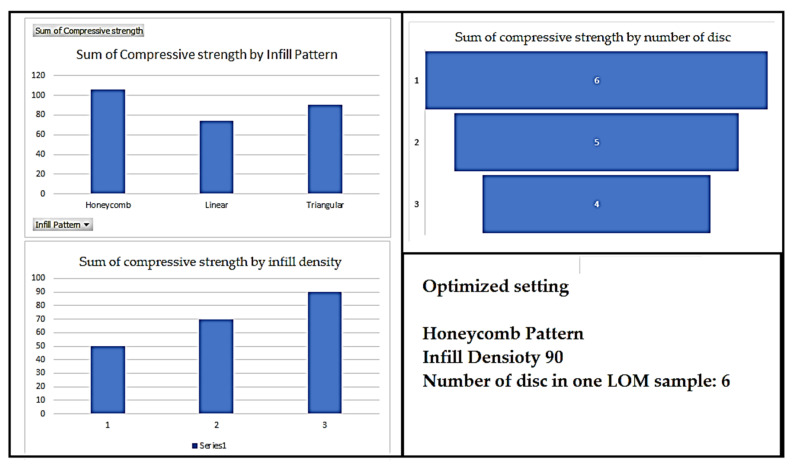
Analysis of the sum of compressive strength values for different input parameters using data from Table 4.

The values obtained in Table 4 are used for the optimization of the 3D printing conditions. From the analysis using data from Table 4, it may be observed that, for the sum of the compressive strength values, infill density of 90%, honeycomb pattern, and 6 discs in a single specimen, are the optimized condition for the LOM manufactured compression samples (see Figure 3). As per the reported Table 4, the best sample held 70% infill density whereas the other optimized conditions (number of discs and infill pattern) were similar to the optimized conditions. The possible reason for this behavior may be due to infill density may have not played important role in deciding the compression behavior.

But, as per the previous reported studies infill density is one of the important criteria for mechanical properties [7,8,9]. In place of infill density, the number of discs has played a vital role in compression strength. As the disc was of small size i.e., 12.7× individual height of each sample, this may have resulted in a decrease in volume inside the samples as various factors contribute to the volume such as the number of upper and lower layers, number of perimeters, infill density, etc. Table 5 shows the fisher value, p-value and percentage contribution of the input parameters towards the compressive strength which has also highlighted that infill density has the least role to play in compression samples for this particular study as the number of upper and lower layers supersedes the infill density processing parameter. Therefore, the influence of the number of upper and lower layers is greater than the infill density.

### 3.2. Stage 2: Compression Properties for Heat-Treated Specimens

**Figure 4:**
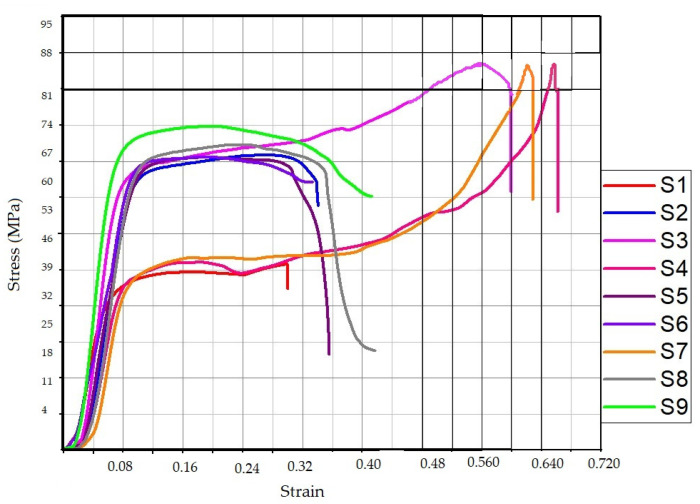
Stress vs strain diagram for the heat-treated samples of stage 2.

From the compression testing of stage 1, it was ascertained that sample 8 with processing parameters ID:70%; IP: Honeycomb, and NoD: 6 was the best and optimized condition for LOM manufacturing of 3D-printed samples. Further at stage 2, the samples were again 3D-printed using DOE Table 3 and were tested for compression behavior. Table 6 shows the compression properties obtained for heat-treated samples. The obtained results have indicated improved compressive strength. This may be due to the reduced thermal stresses, improved internal structure, and reduction in void percentage to the total volume by post-heat treatment of the samples. Figure 4 shows the stress vs strain behavior of the tested sample for stage 2. From Table 6 it may be observed that samples 3, 4, and 7 held compression strengths of 87.24, 87.07, and 87.10 MPa respectively. Among these samples, the third sample has shown maximum compressive strength but the corresponding strain value for sample 3 was 0.59. Whereas, sample 4 held a maximum strain value of 0.65 for the corresponding strength of 87.07 MPa.

This behavior for the heat-treated sample may be explained based on the selected DOE for stage 2. Sample 3 was heat treated at a temperature of 65 °C for 10 min, and sample 4 was heat treated for 20 min at a temperature of 55 °C. Whereas sample 7 was heat treated with a temperature of 55 °C for 30 min time intervals. The results have indicated that high temperature and low time value (sample 3: Temperature: 65 °C, 10 min interval) work equally well for low-temperature value and high time interval (Sample 7: 55 °C, 30 min). The least effect in compressive strength has been observed for sample 1 with heat treatment at a temperature of 55 °C and a time interval of 10 min. Thus, it can be established that low temperatures and low time intervals for heat treatment have resulted in insignificant results.

This may be due to the reason that low temperature and low time interval were unable to change any of the internal characteristics (void reduction, reduction in thermal stresses in 3D-printed layers) of the 3D-printed sample. Similarly, sample 9 was heat treated at 65 °C for a time interval of 30 min which has not given significant improvement in strength indicating that high temperature with high time interval is also not good for the heat-treated samples. This may be due to the reason that the samples were exposed to the high-temperature value above the glass transition temperature (T_g_) of the polymer for a prolonged time which may have induced brittleness in the sample when cooled in air.

#### Regression Analysis for Stage 2

**Figure 5:**
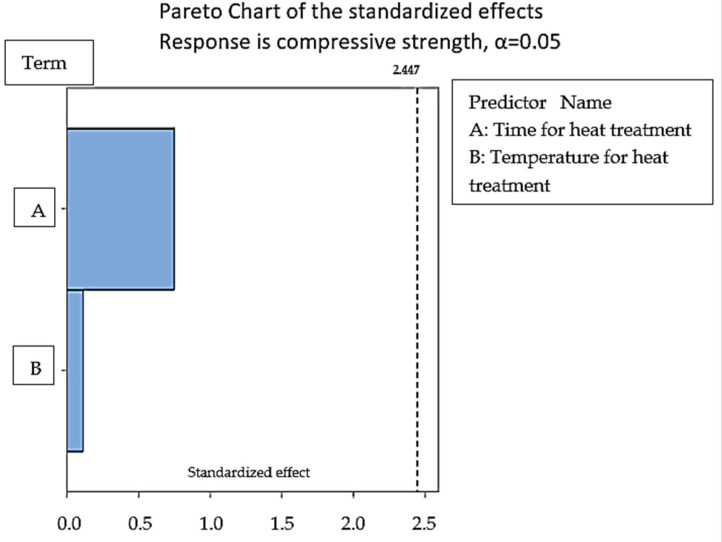
Pareto chart model for regression analysis of the sample for stage 2.

**Figure 6:**
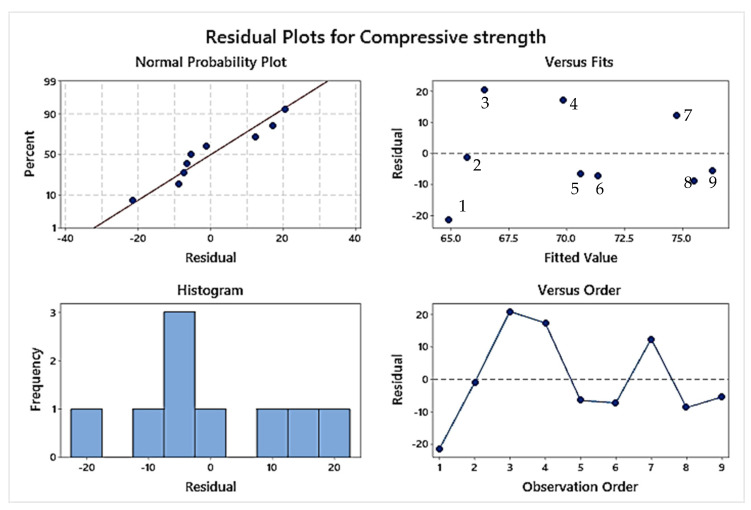
Residual plot for compressive strength of samples of stage 2.

The results obtained by compression testing for stage 2 samples are further used for regression analysis to plot an equation driving the relation of compressive strength with the selected input parameters of heat treatment. Equation (2) obtained by regression analysis indicated that the time for heat treatment has a greater role to play than the temperature range as the selected range of temperatures 55 °C, 60 °C, and 65 °C are near to the Tg value of PLA. The second parameter time of heat treatment has thus affected the material properties largely. Figure 5 shows the Pareto charts for the standardized effect of compression strength which also highlighted the greater effect of the time parameter in the case of polymeric heat treatment. Figure 6 shows regression four in one chart which shows the data used for the regression analysis obtained by compression testing is normal and follows the normal trend line. From Figure 6 the residual vs fit graph shows that the value for sample 3 was the highest but is away from the zero line. The compressive strength values for samples 8 and 9 were near the zero-base line which suggested that the higher time and temperature values favored the compressive strength for the heat-treated samples.

(2) Compressive strength=51.5+0.492 Time for heat treatment+0.16 Temperature for heat treatment

### 3.3. Comparison of the Heat-Treated and Non-Heat-Treated Morphological Properties

**Figure 7:**
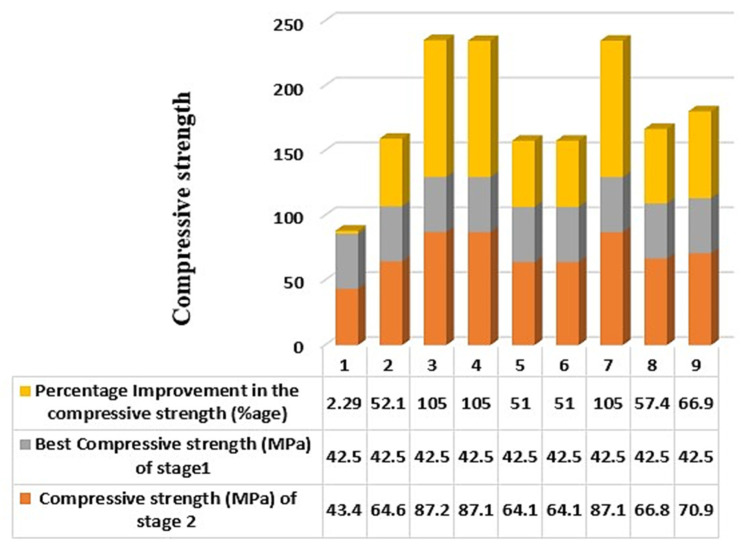
Comparison graph for the best sample of stage 1 (sample 8) and samples of stage 2.

The obtained results for stage 1 and stage 2 were compared further to observe the effect of heat treatment on LOM-manufactured 3D-printed samples. The comparison in Table 7 highlights the difference in the compressive strength for normal and heat-treated samples. From the comparison in Table 7, it has been ascertained that the maximum improvement in the compression strength is for samples 3, 4, and 7, respectively. The compressive strength has increased significantly more than 100% for samples 3, 4, and 7. The results of stage 2 are compared to the compressive strength for the sample 8 results of stage 1 which was found highest among all from stage 1. A minimum change in percentage has been observed for sample 1 equivalent to 2.29%. Figure 7 shows the percentage change in compressive strength for the best sample of stage 1 with the samples of stage 2.

## 4. Morphological Results

### 4.1. SEM Analysis for Stage 1

**Figure 8:**
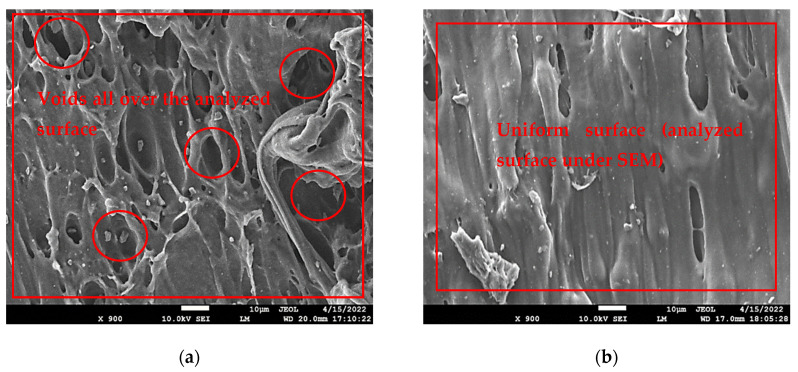
SEM micrographs for (**a**) sample 3 and (**b**) sample 8 at ×900.

The samples of stage 1, sample 8, and sample 3 were compared to each other as sample 3 and sample 8 were obtained as the poorest (21.91 MPa) and best sample (42.47 MPa) from stage 1 as per Table 4. From the SEM images for samples 3 and 4 it may be observed that sample 8 held a more uniform surface texture than sample 8 and the voids over the selected surface area were also not significant. Figure 8a,b show the SEM image for sample 3 and sample 8, respectively. Sample 3 has a comparatively large number of voids when compared to sample 8’s SEM image which signified that the different 3D printing parameters have resulted in different internal characteristics. Sample 3 contained four discs in one sample which comprised 32 upper and lower layers and 50% infill density which resulted in the low strength of the sample as observed in compressive strength for the samples (see Table 4). 

### 4.2. SEM Analysis for Stage 2

**Figure 9:**
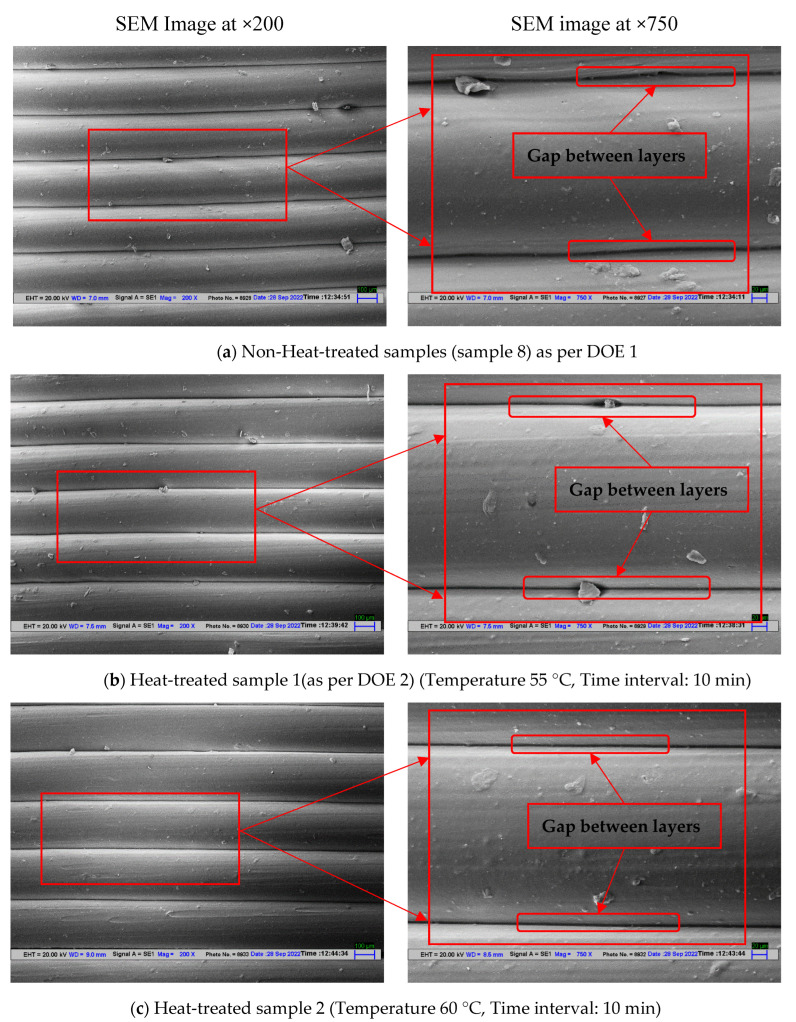

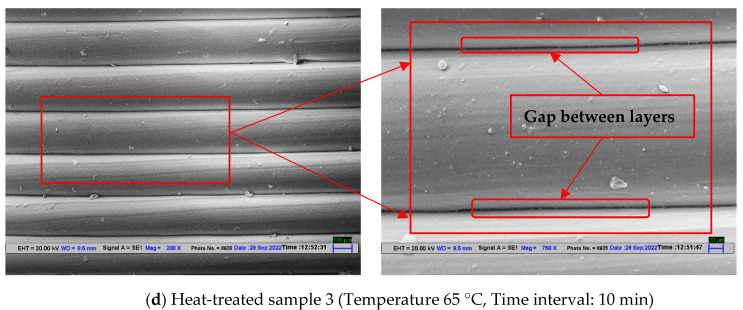
SEM Images for (**a**) non-heat-treated sample, (**b**) sample 1 as per DOE 2, (**c**) sample 2 as per DOE 2, (**d**) sample 3 as per DOE 2 at ×200 and ×750.

Further, among samples in stage 2, four samples were selected for SEM analysis. To study the effect of heat treatment one non-heat-treated sample from stage 1 and 3 heat-treated samples from stage 2 were analyzed. The three heat-treated samples 1 (55 °C, 10 min), sample 2 (60 °C, 10 min), and sample 3 (65 °C, 10 min) as per DOE 2 were selected so that a clear understanding can be made of the effect of heat treatment on 3D-printed polymeric samples of PLA. From SEM analysis of the not heat-treated sample, it has been observed that the gap between the adjacent layer was significant as highlighted with arrows and marking in Figure 9a. The samples with heat treatment under different conditions have observed an effect on layer gaps. It has been observed that with increasing temperature the layer gap was suppressed/reduced resulting in better joining of the adjacent layer ultimately resulting in better compressive strength. As it is evident from Figure 9b SEM image for sample 1 as per DOE 2 (heat treated with 55 °C temperature condition for a low time interval of 10 min) has an insignificant difference (slight difference in layer gap) with the sample which was not heat treated. This supports the mechanical observation from the comparison in Table 7. It has been observed that sample 1 as per DOE 1 (heat treated with temperature 55 °C for 10 min) has an insignificant difference in compressive strength (2.29% increase in compressive strength) from sample 8 of stage 1. 

### 4.3. SEM Analysis for the Compressed Samples of Stage 2 

**Figure 10:**
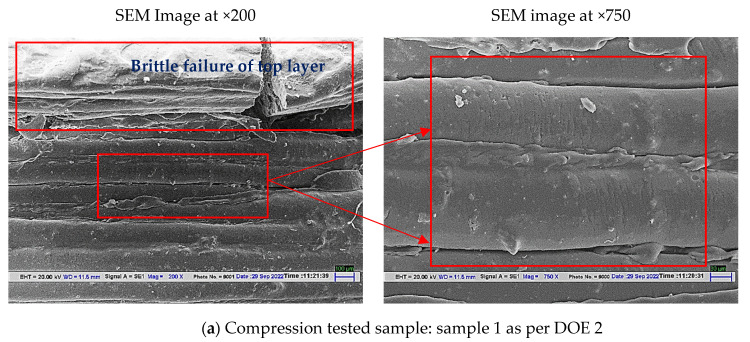

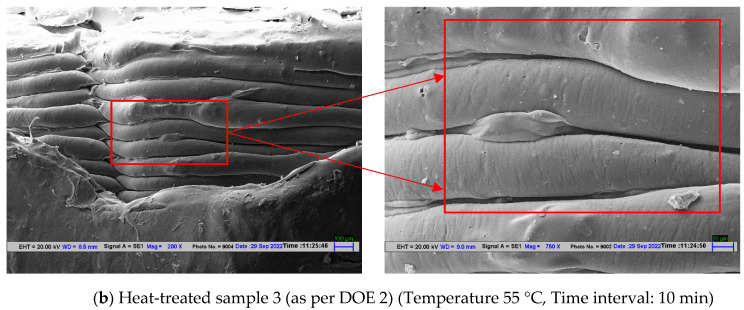
SEM images for compression-tested samples (**a**) sample 1 and (**b**) sample 3.

The compression-tested samples of stage 2, sample 3 (best among all), and sample 1 (worst among all) were also analyzed under SEM after cutting the compressed sample from half-plane. The results for sample 1 indicated that sample 1 held brittle failure as the upper layer was torn off due to compressive load (see Figure 10a). Sample 1 which was heat treated for low temperature and low time interval had hardly any effect on the material properties. But sample 3 which was heat treated for 10 min at 65 °C may have experienced low internal thermal stresses of the structure developed due to 3D printing. Thus, low internal stresses of polymeric structure may have resulted in low brittleness or stiffness and therefore sample 3 with low internal thermal stresses and stiffness resulted in ductile failure than brittle failure, and no signs of brittle tearing of layers were observed on the sample (see Figure 10b).

## 5. Conclusions

The work explored the effect of heat treatment on the polymeric samples manufactured through the LOM route. A comparison of the non-heat-treated and heat-treated samples was conducted to understand the rationale behind the changes observed in the mechanical and morphological characteristics of the sample. The following are the conclusions of the different stages.

Remarks of stage 1: 3D printing of samples using Infill density: 70%, Infill pattern: honeycomb, and six discs in a single LOM manufactured sample may be concluded as the optimized condition for stage 1 with a compression strength of 42.47 MPa. The optimized condition has shown better mechanical strength compared to sample 1 and the results are supported by SEM analysis.

Remarks of stage 2:

(a) The heat-treated samples have shown significant improvement in compression strength and strain values. Sample 3 has shown maximum compressive strength but the corresponding strain value for sample 3 was 0.59.
(b) From the heat treatment analysis, it may be concluded that high temperature and low time value (sample 3: temperature: 65 °C, 10 min interval) work equally well for low-temperature value and high time interval (Sample 7: 55 °C, 30 min). The least effect in compressive strength has been observed for sample 1 with heat treatment at a temperature of 55 °C, and time interval of 10 min.
(c) Morphological analysis through SEM supported the mechanical observation. The heat treatment of the samples has improved the interlayer bonding by reducing the gap between the adjacent layers.

Remarks of comparative analysis

(a) From the comparison of heat-treated and non-heat-treated samples, it may be concluded that the heat treatment at a high temperature near Tg for a low time interval of 10 min improved the compressive strength by 105.42%.
(b) Morphological analysis using SEM characterization has supported the observed behavior.

**Future study**

The study has found that the post-heat treatment of the FDM-printed samples resulted in better mechanical and morphological characteristics. Further investigation can be made for a wide range of input parameters of FDM printing. The current study was limited to compression properties of single-material FDM-printed LOM-manufactured samples; therefore, a similar study may be explored for bending, impact, and other mechanical properties. In future works, multi-material components may be joined using the proposed technique for improved results.

## Figures and Tables

**Table 1:** Summarized literature review highlighting effort made and findings of the work.

S.No.	Research Group’s	Effort Made	Findings
1	Yarahmadi et al. [7]	Heat-treated polyvinyl chloride (PVC) for the selected range of temperature between 55 °C to 165 °C over a period of time 1 h to 143 days.	The work observed a decrease in break elongation with an increase in temperature above 55 °C and for increasing aging time.
2	Fouad et al. [8]	Post-heat treatment of ultra-high molecular weight polyethylene (UHMWPE) polymeric material at 50 °C, 80 °C, and 100 °C for 2 to 4 h	Improvement in the fracture strength, modulus, and yield strength of the tensile specimen significantly in the range of 11 to 25% for different material characteristics.
3	Pagno et al. [9]	Studied the effect of heat treatment on PBAT and PCL reinforce matrix for membrane application in drug removal.	Improved the retention capacity of electro-spun-produced membranes by 67%.
4	Chalgham et al. [10]	Heat treatment of PLA for orthosis application.	Heat treatment of samples led to a change in geometry for orthosis without decreasing the maximum force.
5	Jayanth et al. [11]	Chemical treatment of ABS after FDM printing.	(a) Decrease in tensile strength of the ABS samples when chemically treated. (b) The surface finishes improved.
6	Storck et al. [12]	Tested different materials of FDM printing for elevated temperatures −40 °C to +80 °C by selecting cycles of 90 min.	PLA material was observed to be stable and other materials lost their dimensional and mechanical characteristics.
7	Zhao et al. [13]	Post heat treatment process effect for PEEK for the temperature of 150 °C to 250 °C (above glass transition temperature Tg) and time interval of 2 h.	The tensile strength for the PEEK samples was observed to be increasing with increasing temperature.
8	Wang and Zou [14]	Observed the effect of heat treatment on short and continuous fiber-reinforced PEEK material matrix.	Improvement in the layer adhesion and diffusion of the adjacent layers leads to better mechanical strength.
9	Wach et al. [14]	Explored the thermal annealing process for PLA matrix to improve the crystallinity degree.	11 to 20% improvement in the PLA strength.
10	Li et al. [16]	Worked on sanding or plasma treatment of the 3D-printed PEEK, PEEK/CF material.	The study indicated a significant increase in the shear strength of the lap-jointed 3D-printed workpiece.
11	Cerezo et al. [17]	Explored heat treatment using a ceramic powder mold	Reduced 80% to 90% chances of deformation in geometry especially in the length of the FDM-printed samples when heat treated with ceramic mold
12	Akhoundi et al. [18]	Studied the impact of the heat treatment annealing process on FDM-printed parts of PLA.	32% increase in the mechanical strength for the heat-treated samples.
13	Guduru and Srinivasu [19]	Explored post-heat treatment and chemical treatment process on PLA/CF reinforced FDM-printed samples.	Significant increase in the tensile strength of PLA/CF material for optimized sample using chemical treatment (80 MPa) and heat treatment (74 MPa) route than normal PLA/CF FDM-printed matrix (70 MPa).
14	Mohammad, et al. [20]	made effort towards 4D printing of PET-G [20] and tried to give material self-healing characteristics	Observed that 75 °C temperature is the optimized range where material exhibits self-healing from bending. Below and above this temperature, the material has shown the least effect on self-healing and shape recovery.
15	Ehrmann et al. [21]	The post-heat treatment process for PLA material for different grades and colors of PLA	The reported results suggested that there are contradictions and low reproducibility of the results with the previously reported data
16	Jayswal and Adanur [22]	The post-heat treatment process for PLA material for textile application	Improved mechanical properties of PLA. Furthermore, more crystallinity was found in the case of heat-treated samples.
17	McLouth et al. [23]	Tested atmospheric plasma treatment (APT) on polyetherimide (PEI)	Observed 35% improved surface and mechanical characteristics of the samples in terms of bond strength. It was also observed that the damaged samples of PEI were repaired at a 100% recovery rate by the applied plasma treatment and bonding technique.
18	Moradi et al. [24]	Exploration for annealing process on PLA samples using laser cutting setup.	The study reported optimized processing conditions for the post-processing as focal position: 0.53 mm, laser cutting speed: 1.19 mm/s, and laser power 36.49 W.
19	Sardinha et al. [25]	proposed a new method called “Ironing” which deals with heating the stacked layer at a defined level while FDM printing.	The results of the study have suggested a 60% improvement in the surface roughness value.
20	Shaik et al. [26]	In-situ pressure technique while FDM printing	10 bar of in-situ pressure improves the mechanical properties by 100–150%.
21	Mazlan et al. [27]	Blow cold vapor treatment of FDM-printed PLA parts	Improved surface characteristics of PLA samples
22	Rafie et al. [28]	In-Situ and vacuum oven-based heat treatment of PLA sample	vacuum oven-based heat treatment of PLA sample proved to be better than in-situ treatment.

**Table 2:** DOE for FDM Printing of compression samples at the first stage.

S.No.	Parameter	Description	Level
1	Infill Pattern	The geometric pattern inside in form of fill	(a) Linear (b) Triangular (c) Honeycomb
2	Infill Density (%age)	Total fill percentage inside the 3D-printed samples	(a) 50 (b) 70 (c) 90
3	Number of discs	Number of discs joined by LOM manufacturing to prepare a standard sample of 25.4 mm as per ASTM D695	(a) 6 (Disc Thickness:3.4 mm) (b) 5 (Disc Thickness:4.28 mm) (c) 4 (Disc Thickness:5.6 mm)

**Table 3:** DOE for heat treatment of samples prepared at stage 2.

S.No.	Time for Heat Treatment	Temperature for Heat Treatment
1	10	55
2	10	60
3	10	65
4	20	55
5	20	60
6	20	65
7	30	55
8	30	60
9	30	65
Description of the variable for stage 2 (a) Temperature: Temperature for heat treatment (b) Time: Period of heat treatment for different samples		

**Table 4:** Compression property table for LOM manufactured samples.

S.No.	Stress	Strain
1	29.71	0.31
2	22.4	0.24
3	21.91	0.19
4	28.38	0.36
5	25.91	0.28
6	36.06	0.37
7	30.38	0.32
8	42.47	0.41
9	32.75	0.34

**Table 5:** Fisher and p-value for the non-heat-treated compression-tested samples of PLA.

Source	F	P	Percentage Contribution
IP	206.41	0.005	51.60
ID	0.48	0.677	0.11
NoD	192.02	0.005	48.29
RE	…	…	…
Total	…	…	…

IP: Infill pattern; ID: Infill density; NoD: Number of discs; RE: Residual error.

**Table 6:** Compression properties for heat-treated samples.

S.No.	F_max_ (N)	dL at F_max_ (mm)	F_break_ (N)	dL at F_break_ (mm)	Strain at Break	Compression Strength (MPa)
1	5500.24	7.48	4484.31	7.50	0.30	43.44
2	8179.14	6.63	6782.61	8.53	0.34	64.60
3	11,045.73	14.96	10,168.34	14.98	0.59	87.24
4	11,023.91	16.52	9221.12	16.54	0.65	87.07
5	8118.13	4.83	2658.78	8.89	0.35	64.12
6	8121.15	4.59	7427.25	8.32	0.33	64.14
7	11,028.00	15.69	6954.72	15.71	0.62	87.10
8	8461.87	5.76	2758.83	10.42	0.41	66.83
9	8973.84	5.03	7032.76	10.31	0.41	70.88

**Table 7:** Comparison table for the best sample of stage 1 with the samples of stage 2.

S.No.	Compressive Strength (MPa) of Stage 2	Best Compressive Strength (MPa) of Stage1	Percentage Improvement in the Compressive Strength (%age)
1	43.44	42.47	2.29
2	64.60	42.47	52.11
3	87.24	42.47	105.42
4	87.07	42.47	105.01
5	64.12	42.47	50.97
6	64.14	42.47	51.03
7	87.10	42.47	105.09
8	66.83	42.47	57.36
9	70.88	42.47	66.89

## Data Availability

Not Applicable.

## References

[1] Khan M.S., Abdul-Latif A., Koloor S.S.R., Petrů M., Tamin M.N. (2020). Representative Cell Analysis for Damage-Based Failure Model of Polymer Hexagonal Honeycomb Structure under the Out-of-Plane Loadings. Polymers.

[2] Mlýnek J., Petrů M., Martinec T., Koloor S.S.R. (2020). Fabrication of High-Quality Polymer Composite Frame by a New Method of Fiber Winding Process. Polymers.

[3] Wong K.J., Johar M., Koloor S.S.R., Petrů M., Tamin M.N. (2020). Moisture Absorption Effects on Mode II Delamination of Carbon/Epoxy Composites. Polymers.

[4] Kashyzadeh K., Koloor S.R., Bidgoli M.O., Petrů M., Asfarjani A.A. (2021). An Optimum Fatigue Design of Polymer CompositeCompressed Natural Gas Tank Using Hybrid Finite Element-Response Surface Methods. Polymers.

[5] Kumar S., Singh I., Koloor S.S.R., Kumar D., Yahya M.Y. (2022). On Laminated Object Manufactured FDM-Printed ABS/TPU Multimaterial Specimens: An Insight into Mechanical and Morphological Characteristics. Polymers.

[6] Koloor S., Abdullah M., Tamin M., Ayatollahi M. (2019). Fatigue damage of cohesive interfaces in fiber-reinforced polymer composite laminates. Compos. Sci. Technol..

[7] Yarahmadi N., Jakubowicz I., Hjertberg T. (2003). The effects of heat treatment and ageing on the mechanical properties of rigid PVC. Polym. Degrad. Stab..

[8] Fouad H., Mourad A.-H., Barton D. (2005). Effect of pre-heat treatment on the static and dynamic thermo-mechanical properties of ultra-high molecular weight polyethylene. Polym. Test..

[9] Pagno V., Módenes A.N., Dragunski D.C., Fiorentin-Ferrari L.D., Caetano J., Guellis C., Gonçalves B.C., dos Anjos E.V., Pagno F., Martinelli V. (2020). Heat treatment of polymeric PBAT/PCL membranes containing activated carbon from Brazil nutshell biomass obtained by electrospinning and applied in drug removal. J. Environ. Chem. Eng..

[10] Chalgham A., Ehrmann A., Wickenkamp I. (2021). Mechanical Properties of FDM Printed PLA Parts before and after Thermal Treatment. Polymers.

[11] Jayanth N., Senthil P., Prakash C. (2018). Effect of chemical treatment on tensile strength and surface roughness of 3D-printed ABS using the FDM process. Virtual Phys. Prototyp..

[12] Storck J.L., Ehrmann G., Güth U., Uthoff J., Homburg S.V., Blachowicz T., Ehrmann A. (2022). Investigation of Low-Cost FDM-Printed Polymers for Elevated-Temperature Applications. Polymers.

[13] Zhao Y., Zhao K., Li Y., Chen F. (2020). Mechanical characterization of biocompatible PEEK by FDM. J. Manuf. Process.

[14] Wang P., Zou B. (2022). Improvement of Heat Treatment Process on Mechanical Properties of FDM 3D-Printed Short- and Continuous-Fiber-Reinforced PEEK Composites. Coatings.

[15] Wach R.A., Wolszczak P., Adamus-Wlodarczyk A. (2018). Enhancement of Mechanical Properties of FDM-PLA Parts via Thermal Annealing. Macromol. Mater. Eng..

[16] Li W., Sang L., Jian X., Wang J. (2020). Influence of sanding and plasma treatment on shear bond strength of 3D-printed PEI, PEEK and PEEK/CF. Int. J. Adhes. Adhes..

[17] Lluch-Cerezo J., Benavente R., Meseguer M., García-Manrique J. (2021). Effect of a Powder Mould in the Post-Process Thermal Treatment of ABS Parts Manufactured with FDM Technology. Polymers.

[18] Akhoundi B., Nabipour M., Hajami F., Shakoori D. (2020). An Experimental Study of Nozzle Temperature and Heat Treatment (Annealing) Effects on Mechanical Properties of High-Temperature Polylactic Acid in Fused Deposition Modeling. Polym. Eng. Sci..

[19] Guduru K., Srinivasu G. (2020). Effect of post treatment on tensile properties of carbon reinforced PLA composite by 3D printing. Mater. Today: Proc..

[20] Aberoumand M., Soltanmohammadi K., Soleyman E., Rahmatabadi D., Ghasemi I., Baniassadi M., Abrinia K., Baghani M. (2022). A comprehensive experimental investigation on 4D printing of PET-G under bending. J. Mater. Res. Technol..

[21] Ehrmann G., Brockhagen B., Ehrmann A. (2021). Shape-Memory Properties of 3D Printed Cubes from Diverse PLA Materials with Different Post-Treatments. Technologies.

[22] Jayswal A., Adanur S. (2022). Effect of heat treatment on crystallinity and mechanical properties of flexible structures 3D printed with fused deposition modeling. J. Ind. Text..

[23] McLouth T.D., Gustafson S.M., Kim H.I., Zaldivar R.J. (2021). Enhancement of FDM ULTEM^®^ 9085 bond strength via atmospheric plasma treatment. J. Manuf. Process..

[24] Moradi M., Moghadam M.K., Shamsborhan M., Bodaghi M., Falavandi H. (2020). Post-Processing of FDM 3D-Printed Polylactic Acid Parts by Laser Beam Cutting. Polymers.

[25] Sardinha M., Vicente C.M., Frutuoso N., Leite M., Ribeiro R., Reis L. (2021). Effect of the ironing process on ABS parts produced by FDM. Mater. Des. Process. Commun..

[26] Shaik Y.P., Schuster J., Katherapalli H.R., Shaik A. (2022). 3D Printing under High Ambient Pressures and Improvement of Mechanical Properties of Printed Parts. J. Compos. Sci..

[27] Mazlan S.N.H., Alkahari M.R., Ramli F.R., Maidin N.A., Sudin M.N., Zolkaply A.R. (2018). Surface finish and mechanical properties of FDM part after blow cold vapor treatment. J. Adv. Res. Fluid Mech. Therm. Sci..

[28] Rafie M., Marsilla K.K., Hamid Z., Rusli A., Abdullah M. (2020). Enhanced mechanical properties of plasticized polylactic acid filament for fused deposition modelling: Effect of in situ heat treatment. Prog. Rubber, Plast. Recycl. Technol..

[29] Elsheikh A. (2022). Bistable Morphing Composites for Energy-Harvesting Applications. Polymers.

[30] Elsheikh A.H., Panchal H., Shanmugan S., Muthuramalingam T., El-Kassas A., Ramesh B. (2022). Recent progresses in wood-plastic composites: Pre-processing treatments, manufacturing techniques, recyclability and eco-friendly assessment. Clean. Eng. Technol..

[31] (2010). ASTM D695-15.

[32] Karimzadeh A., Ayatollahi M.R., Koloor S.S.R., Bushroa A.R., Yahya M.Y., Tamin M.N. (2019). Assessment of Compressive Mechanical Behavior of Bis-GMA Polymer Using Hyperelastic Models. Polymers.

[33] Khalajmasoumi M., Koloor S., Arefnia A., Ibrahim I., Yatim J.M. (2012). Hyperelastic Analysis of High Density Polyethylene under Monotonic Compressive Load. Appl. Mech. Mater..

